# Medical Thoracoscopy for Undiagnosed Exudative Pleural Effusion: Experience from Two Tertiary Care Hospitals of Nepal

**DOI:** 10.31729/jnma.4873

**Published:** 2020-03-31

**Authors:** Bishow Kumar Shrestha, Shital Adhikari, Binay Kumar Thakur, Dipen Kadaria, Kishor Kumar Tamrakar, Mukti Devkota

**Affiliations:** 1 Pulmonary, Critical Care and Sleep Medicine Unit, Chitwan Medical College Teaching Hospital, Chitwan, Nepal; 2 Department of Thoracic Surgery, B P Koirala Memorial Cancer Hospital, Chitwan, Nepal; 3 Division of Pulmonary, Critical Care and Sleep Medicine, University of Tennessee HSC, Memphis, TN, USA; 4 Department of Surgery, Chitwan Medical College Teaching Hospital, Chitwan, Nepal

**Keywords:** *exudative pleural effusion*, *tertiary care hospitals*, *thoracoscopy*

## Abstract

**Introduction:**

Medical thoracoscopy has recently gained renewed interest due to its minimal invasive nature and high yield diagnostic outcome. This study aims to observe diagnostic yield and safety of medical thoracoscopy in undiagnosed exudative pleural effusion.

**Methods:**

This is a descriptive cross-sectional study conducted in two tertiary care hospitals in Chitwan from March 2018 to May 2018. Ethical approval from the Institutional Review Board was obtained. Convenient sampling was done that included all the patients who met criteria for undiagnosed exudative pleural effusion after diagnostic thoracocentesis. Patients having contraindication to procedure and who refused consent were excluded. Statistical analysis was performed using IBM SPSS Statistics 20 and data are presented as mean SD and frequency (percentage).

**Results:**

A total of 14 patients underwent rigid medical thoracoscopy. All 14 patients had unilateral pleural effusion. The overall diagnostic yield was 100%. Malignancy was the most frequent histopathology diagnosis seen in 11 (78.57%) patients, the commonest being metastatic adenocarcinoma in 8 (57.1%). Pleural tuberculosis and acute-on-chronic pleuritis were seen in 2 (14.3%) and 1 (7.1%) patients, respectively. Pleural deposits and hemorrhagic pleural fluid were the two commonest findings, seen in 10 (70.1%) and 9 (64.3%) patients, respectively. Two (14.3%) patients clinically treated as tuberculous pleural effusion was re-diagnosed to have metastatic adenocarcinoma. Procedure related mortality and major complications were nil. Common procedurerelated minor complications observed were mild to moderate pain and mild bleeding, observed in 3 (21.4%) and 2 (14.3%) patients, respectively.

**Conclusions:**

Medical thoracoscopy is a safe, well-tolerated and high yield procedure in undiagnosed exudative pleural effusion. This art of medicine should be promoted in daily medical practice.

## INTRODUCTION

Medical thoracoscopy (MT) allows visualization of pleural cavity and permits biopsy of target pleural lesions under direct visual guidance. Therapeutic interventions like chemical pleurodesis can also be performed in the same sitting.^1^ It is a relatively safe and minimally invasive procedure which can be performed under local anaesthesia and/or conscious sedation using rigid or semi-rigid thoracoscopes.^2^ MT has been shown to have higher diagnostic yield ranging from 86.2% to 100% in unexplained exudative pleural effusion (PF).^3–6^.

Despite high diagnostic accuracy, simpler sedation protocol and safety, MT has not gained wider acceptability even in developed countries. MT could have major role in underdeveloped countries like Nepal where access to video assisted thoracic surgery (VATS) and open thoracic surgery/biopsy is limited due to lack of thoracic surgeons, high cost and lack of specialized surgical centres.

In this study, we aim to observe diagnostic yield and safety and show the importance and impact of MT in evaluation of undiagnosed pleural effusion.

## METHODS

This descriptive cross-sectional study was conducted over a period of three months (March 2018-May 2018) in Chitwan Medical College Teaching Hospital (CMCTH), Chitwan and B P Koirala Memorial Cancer Hospital (BPKMCH), Chitwan. Ethical clearance was obtained from Institutional Review Board (Ref: CMCIRC/075/076-121). Convenient sampling for the period of three months was done. All the participants were evaluated in detail with clinical history, physical examination, laboratory investigations including hemogram, renal and liver function tests, coagulation profile, viral markers, sputum analysis, electrocardiogram and CT scan of the chest. PF analysis was done for cell counts, sugar, protein, lactate dehydrogenase, adenosisne deaminase, Gram's stain, acid fast bacillus smear, culture and cytopathology. Fourteen patients with results of exudative PF by Light's criteria but without other etiologic diagnosis were included. Two patients were excluded; one who refused to go for further treatment due to terminal illness and the other who had contraindication to the procedure due to uncorrected coagulation disorder (prothrombin time more than 25 sec compared to control and platelet count of 45,000/cumm). A written informed consent was obtained from all the participants of the study. All the findings were entered in the proforma.

All the MT procedures were performed in operating room (OR). The patients were made to lie in lateral decubitus position with healthy side down and arms above the head. In OR rib spaces evaluation, quantification of pleural fluid, presence of adhesions in the pleural cavity and entry point selection was done using chest ultrasound. The point of entry was selected as the point of maximum fullness that was free of adhesions below in the pleural cavity, in the mid-axillary zone between fourth and seventh intercostal space. Double port thoracoscopic procedure was performed using 10 mm rigid telescope (STRYKER 1588 system in both the centres) and 5 mm biopsy forceps. All the procedures were performed under conscious sedation (with midazolam 2 to 8 mg and Fentanyl 50 to 100 mcgm) and local anesthesia (with 2% lidoacine solution infiltration at the incision site) with continuous oxygen supplementation via nasal cannula at 2 to 6L/min, and monitoring of heart rate, electrocardiogram, noninvasive blood pressure and oxyhemoglobin saturation with cardiac monitor and finger probe. All the pleural fluid was suctioned out and an artificial pneumothorax created by allowing air to suck into the pleural space while the patient breathed spontaneously.

Thorough inspection of pleural cavity was done, any abnormalities seen were noted. Pleural adhesions were gently broken down mechanically, where possible. Three to six biopsies were taken with alligator jaw forceps from abnormal parietal pleura using lift and tear technique. The biopsy samples were immediately placed in Formalin container for histopathological analysis. Then lung re-expansion was assessed; three patients in whom lung re-expansion was complete and malignant pleural effusion was contemplated, pleurodesis was performed with diluted Povidone-Iodine solution (20 ml of 10% Povidone-Iodine mixed with 80 ml of Normal Saline) following premedication with 2mg/kg of 2% lidoaince solution in 50 ml Normal Saline. Twenty four Fr Intercostal drainage (ICD) tube was inserted and left in situ prior to shifting the patient out of OR. The time required to complete the procedure was measured from the time when local anaesthetic was infiltrated to the insertion of ICD.

The pleural biopsy specimens were then dispatched for histopathlogical examination along with proper sample identification. Patient was transferred to ward after the effects of sedation disappeared. A chest X-ray was performed after 1 hour and expansion of lung reassessed. Pain due to pleurodesis and procedure was managed with paracetamol and opoid analgesics. The ICD was removed when pleural fluid drainage was <150 ml/24 hours.

All the pleural biopsy samples were examined and interpreted by the pathologists of the respective hospitals. Histopathology reports that mentioned non-specific pleuritis (like acute-on-chronic pleuritis) was taken as positive yield and included for yield calculation. The throacoscopic findings, complications, duration of procedure and ICD, hospital stay, condition at discharge and histopathology reports were recorded in the proforma. The data collected were entered and analyzed using IBM SPSS Statistics 20 and presented as meanSD and frequency (percentage). Diagnostic yield was defined as percentage of positive diagnoses out of throacoscopic pleural biopsy specimens from all the patients.

## RESULTS

A total of 14 MT were performed during the study period. Table 1 shows the baseline characteristic of the patients.

**Table 1:** Baseline Characteristics.

Variables	n (%)
Age (years)	64 (36 79)
Sex	
Male	8 (57.1)
Female	6 (42.9)
Comorbidities	
Yes	6 (42.9)
No	8 (57.1)
Smoking status	
Current smoker	5 (35.7)
Ex-smoker	4 (28.6)
Non-smoker	5 (35.7)
Alcohol consumer	6 (42.9)
Chief complaints (present for > 1 month)	
Dyspnea	12 (85.7)
Chest pain	11 (78.6)
Cough	8 (57.1)
Hemoptysis	4 (28.6)
Weight loss	4 (28.6)
Hoarseness of voice	1 (7.1)
Radiological (CT scan) findings	
Pleural effusion	14 (100.0)
Mild	4 (28.6)
Moderate	6 (42.9)
Gross	4 (28.6)
Laterality of Pleural Effusion	
Right Side	8 (57.1)
Left Side	6 (42.9)
(All 14 cases had unilateral pleural effusion)	
Passive collapse	6 (42.9)
Pleural deposits	4 (28.6)
Lung mass	4 (28.6)
Nodular parenchymal infiltration	1 (7.1)
Lobar consolidation	1 (7.1)
Pleural fluid characteristics on thoracocentesis	
Hemorrhagic	9 (64.3)
Straw coloured	4 (28.6)
Serous	1 (7.1)
Diagnostic thoracocentesis prior to medical thoracoscopy	
One diagnostic thoracocentesis	8 (57.1)
Two diagnostic thoracocentesis	6 (42.9)

All the patients had negative sputum culture/sensitivity, AFB microscopy and cytology. All the patients had unilateral pleural effusion; 8 (57.1%) had right pleural effusion and 6 (42.9%) had left pleural effusion. Similarly, 8 (57.1%) patients had one pre-thoracoscopic diagnostic thoracocentesis and 6 (42.1%) patients had two pre-thoracoscopic diagnostic thoracocentesis. Light's criteria for exudative pleural effusion were fulfilled in all the pleural fluid samples. The most common thoracoscopic finding was pleural deposits, seen in 10 (71.4%) patients (Table 2).

**Table 2:** Thoracoscopic findings n (%)

Pleural Deposits	10 (71.4)
Parietal pleura	7 (50.0)
Parietal and visceral pleura	2 (14.3)
Parietal and Diaphragmatic pleura	1 (7.1)
Inflamed parietal pleura	4 (28.6)
Pleural thickening	3 (21.3)
Localized	1 (7.1)
Diffusely thickened parietal pleura	1 (7.1)
Diffusely thickened parietal and visceral pleura	1 (7.1)
Pleural adhesions	3 (21.4)
Flimsy adhesion	2 (14.3)
Dense adhesion	1 (7.1)
Sago-grain like granules (Parietal Pleura)	1 (7.1)
Median volume of fluid (ml)	1500 (400 2800)

Histopathological diagnosis was made in all 14 patients after thoracoscopy and pleural biopsy, resulting in a net diagnostic yield of 100.0 %. Out of 14 patients, 11 (78.6%) had malignant disease, two (14.3%) had tuberculosis, and one (7.1%) had benign condition (acute-on-chronic pleuritis) as shown (Table 3).

**Table 3:** Pleural biopsy histopathology diagnosis.

Diagnosis	n (%)
Malignancy	
Metastatic adenocarcinoma	8 (57.1)
Metastatic NSCLC	1 (7.1)
Invasive adenocarcinoma	1 (7.1)
Carcinomatous deposits	1 (7.1)
Non-Malignancy	
TB	2 (14.3)
Acute on chronic pleuritis	1 (7.1)

Two patients, who were treated with anti-tubercular therapy on clinical suspicion of TB pleural effusion but were not responsive to the treatment, were found to have metastatic adenocarcinoma by pleural biopsy histopathology.

The most common pleural fluid characteristic was hemorrhagic which was seen in 9 (64.3%) patients, all with malignant conditions (eight metastatic adenocarcinoma, one invasive adenocarcinoma). Straw-colour PF was seen in 4 (28.6%) patients, two (14.3%) of them had tuberculosis by pleural biopsy histopathology. Nine (64.3 %) patients had partial and five (35.7 %) patients had complete lung re-expansion after the procedure. Pleurodesis was performed in three (21.4%) patients with diluted Providence-iodine. Relationship between histopathology diagnoses, pleural fluid characteristic and post-procedure lung reexpansion is shown (Table 4).

**Table 4:** Histopathologic diagnoses and their relationship to pleural fluid characteristic and post-thoracoscopic lung expansion.

	Pleural Biopsy Histopathologic diagnosis
	Metastatic Adeno Ca	T B	Metastatic NSCLC	Invasive Adeno Ca	Acute-onc hronic pleuritis	Carcinomatous deposits
	(n = 8)	(n = 2)	(n = 1)	(n = 1)	(n = 1)	(n = 1)
Pleural fluid characteristics						
Serous						
Straw color	0	0	0	0	1	0
Hemorrhagic	0	2	1	0	0	1
	8	0	0	1	0	0
Post-procedure lung re-expansion					
Incomplete	6	0	1	1	0	1
Complete	2	2	0	0	1	0

Adeno Ca: Adenocarcinoma; TB: Tuberculosis; NSCLC: Non-small cell lung cancer

The most common complication observed was mild to moderate pain in three (21.4%) patients, followed by minor bleeding during the procedure in two (14.3%) patients. Other complications observed were transient hypoxemia and mild fever, each of which was seen in one (7.1%) patients each. Table 5 shows the complications observed.

**Table 5:** Complications observed.

Complication	n (%)
Pain	3 (21.4)
Minor bleeding	2 (14.3)
Hypoxemia	1 (7.1)
Mild fever	1 (7.1)

Average duration for thoracoscopic procedure was 34 minutes (range: 25 to 41 minutes). At the time of discharge, 11 (78.6%) patients had their ICD removed and three (21.4%) patients were discharged with ICD in-situ. Average duration of ICD placement was four days (range 2- 5 days).

## DISCUSSION

MT was very helpful in evaluation of undiagnosed PE in our study. The overall diagnostic yield in this study was 100%, which was comparable to several studies.^3,7–10^ However, our yield was somewhat higher (reached 100%) than that reported by some other studies. The reasons for this high yield could be smaller sample size and all patients having unilateral exudative pleural effusion. MT was shown to have higher yield in unilateral pleural effusion than in bilateral pleural effusion.^11^ Yet another possible reason for this high yield could be due to the inclusion of the benign condition, acute-onchronic pleuritis, in the yield calculation. Investigators had calculated yield in different ways; some had included benign causes like chronic pleuritis,^7,12^ and others had not included them in yield calculation.^13^

In this study, most of the patients had histopathology diagnosis of malignant disease with metastatic adenocarcinoma (57.1%) being the commonest. This finding is in agreement with findings of Hansen et al.^7^ Several factors could have contributed for this result. Our study included patients from tertiary cancer hospital where most of the patients were referred on suspicion of malignancy. In addition, all our patients had all the four clinical clues (symptomatic period, absence of fever, bloody pleural effusion, CT scan chest suggestive of malignancy) that are reported to increase the likelihood of malignant disease.^14^

The second commonest histopathology diagnosis in our study was pleural tuberculosis, which was present in 2 cases (14.3%). This is however somewhat less than what we had expected and that reported by other investigators too.^13^ This could again be due to clustering of patients referred to cancer hospital. These two patients with pleural biopsy histopathology diagnosis of pleural tuberculosis had pleural fluid ADA value less than 30 U/L. And another two patients who were being treated for tuberculous pleural effusion on the basis of lymphocyte predominant PF with high PF ADA levels (PF ADA levels were 65 and 67 U/L), were found have metastatic adenocarcinoma. These observations suggest that PF lymphocytes and ADA level alone would be insufficient to diagnose TB pleural effusion. The unresolved clinically suspicious TB pleural effusion after anti-tuberculosis treatment should hint the possibility of other etiology of pleural effusion. Histopathology diagnosis through pleural biopsy is useful in such cases.

Pleural deposits seen in 10 (70.1%) patients were the most common thoracoscopic finding in our study. Nine patients (64.3%) had hemorrhagic pleural effusion. Malignant pleural invasion is commonly associated with both pleural deposits and hemorrhagic effusion, as reported in several other studies.^9,15^

There were no procedure related mortality or morbidity in our study. These findings are comparable to BTS pleural disease guideline report.^16^ In our study, mild to moderate pain had been reported by three patients (21.4%) and minor bleeding during occurred in two patients (14.3%). These minor complications are slightly higher than that reported in the BTS guideline(17) and by Agarwal et al.^17^ The reason for these slightly increased minor complications could be due to use of rigid thoracoscope (instead of semi-rigid) and double puncture technique in our study. Rigid thoracoscope has the disadvantage of less manoeuvrability, which can be more so when the rib spaces are narrower. Relatively larger biopsy forceps than in semi-rigid thoracoscope, introduced through the second port could have given larger biopsy material,^10^ but at the cost of slightly increased pain and bleeding.

In our study, eight patients (51.7%) right sided and six (42.9%) had left sided pleural effusion. This observation is similar to that observed by Kiani A et al.^18^ The average time for completion of throacosocpic procedure was 34 minutes (range: 25 to 41 minutes), which is closely similar to that reported by Kim SJ et al.^19^ It is to be noted that all the MT procedure performed in this study were diagnostic, the procedure time could have been longer if therapeutic interventions were performed. Five patients had complete expansion of lung after drainage of pleural fluid; pleurodesis using diluted Providence-iodine (Betadine) was performed in three (21.4%) in whom malignant cause of pleural effusion seemed likely, the rest two in whom malignant cause seemed unlikely did not underwent pleurodesis. Providence-iodine had been shown to be cheaper, easily available and effective agent for pleurodesis in several studies with its success rate over 85%.^20–22^

Of the total 14 patients, 11 patients (78.6%) had their intercostal drain (ICD) removed at the time of discharge. The average duration of ICD in these patients was four days (range 2 to 5 days), which is comparable to other studies.^19,23^ Three of our patients were discharged with ICD in situ and they were not followed up, since follow-up was not part of the study protocol. The main limitation of our study was the smaller sample size. If the studies were to be conducted separately in these hospitals, the pattern of pleural biopsy histopatholgy could have been different.

## CONCLUSIONS

Medical thoracoscopy is a relatively safe and minimally invasive procedure with high diagnostic yield and should be considered for both diagnostic and therapeutic purposes especially in all undiagnosed exudative pleural effusion. MT has even more importance and impact in places where access to VATS and open thoracotomy is limited due to various reasons.
